# An Enquiry into the Causes of the Insalubrity of Flat and Marshy Situations; and Directions for Preventing or Correcting the Effects Thereof

**Published:** 1801-12

**Authors:** Wm. Currie


### *An Enquiry into the Causes of the Insalubrity of flat and marshy Situations; and Directions for preventing or correcting the Effects thereof; by Mr.* Wm. Currie.

THAT flat and marſhy ſituations are unfavourable to health, and that intermittent and remittent fevers, with bilious evacuations, are particularly prevalent in ſuch ſituations during the ſeaſon of Autumn in temperate climates as well as within the tropics, has been remarked by phyſicians and hiſtorians in every age.

But although they have agreed with reſpect to the fact, they have differed materially with reſpect to the cauſe of this circumſtance.

A deſire of aſcertaining the true cauſe of this inſalubrity induced me to engage in the enquiry which I am now about to ſubmit to the public; and I hope the time and attention which I have beſtowed upon a ſubject ſo intereſting to mankind, will not be deemed labour miſemployed.

The atmoſphere in ſalutary ſituations, has been demonſtrated by Mr. Lavoiſier and his colleagues, to be a compound body conſiſting of two diſtinct gaſes or aeriform fluids, the one called azote or nitrogen gas, and the other oxygen gas or pure reſpirable air; and that in one hundred parts of the atmoſphere, the proportions of theſe gaſſes are 72 of the azote and 28 of oxygene, or as three to one.

From Mr. Vanbreda's experiments on the atmoſphere of marſhes in the autumnal ſeaſon, which he ſubjected to the common teſt of nitrous air in the eudiometer, it appears that theſe proportions were very different; there being but 14 or 15 parts of oxygen, to 84 or 85 of azote, but that the bulk was ſupplied, and the ſame weight preſerved by a certain quantity of carbonic gas or fixed air, and a ſmall portion of hydrogen and ammoniacal gaſes or aeriform fluids.

All theſe gaſes are the effects of vegetable and animal putrefaction, and muſt be derived from the ſoil, or the vegetable and animal ſubſtances connected with the ſoil.

The ſoil of marſhes is compoſed entirely of vegetable and animal ſubſtances, which have undergone the proceſs of putrefaction, and conſiſts principally of vegetable earth, carbon or charcoal and nitre, mixed with more or leſs calcareous and argillaceous earth, and by diſtillation affords oil, hydrogen, and azote.

From this ſoil, and from the various vegetable and animal ſubſtances mixed with it, and conſtantly putrefying in hot weather, it has been ſuppoſed miaſmata iſſue, which give origin to the diſeaſes peculiar to marſhy ſituations; and as there are no fubftances but thofe gafes, already enumerated, which can be difcovered to iffue from a marfhy foil, or from putrefying vegetable or animal fubftances, if thofe difeafes depend upon miafmata or effluvia, thefe miafmata muft confift of one or more of the gafes enumerated.

In order to determine this matter, it will be neceffary to enquire into the effects which thefe fubftances, fingly or combined, ufually produce on the living human body.

If the carbonic gas or fixed air, when applied in a certain quantity or in a concentrated ftate, deftroys life inftantly by its action on the irritability of the mufcular fibres of the heart, as from the obfervations of Meffrs. Prieftley, Bergman, Fontana, Cavallo, and other philofophers of credit, appears to be the cafe, nothing is more probable than that a lefs quantity, though much weakened by diffufion in, and mixture with, the atmofpheric air, would operate in a fimilar manner, though in a lefs degree, and occafion a difeafe of a paralytic or infenfible kind, and not an intermittent or remittent, fince in thefe laft the fenfibility and irritability are manifeftly increafed.

That the hydrogen gas or inflammable air, has little or no fhare in the generation of the difeafes under confideration, is rendered evident by the experiments of Chaptal, De Rofier, and Beddoes.

No alteration is made in their properties by the mixture of carbonic with hydrogenous gas. No decompofition takes place, no caloric is fet at liberty, or heat rendered fenfible by fuch union. We may therefore, from what has now been ftated, conclude, that neither carbonic nor hydrogen gas, fingly or combined, is the miafma or effluvium by which the difeafes in queftion are produced.

In confequence of the putrefaction of farinaceous plants, and all fuch as abound more in gluten than in the faccharine or mucilaginous principles, as well as from the putrefaction of animal fubftances, an ammoniacal gas is produced, owing to the union of the hydrogen, evolved in the putrefactive fermentation, with the fuperabundant azote of the atmofphere. But this gas, inftead of diminifhing the powers of the human body, is well known to have a contrary effect, except when received into the lungs in a large quantity, and then it proves deftructive from its ftimulating quality, inducing a fpafm on the glottis or bronchiæ. That neither the water of marfhes, nor the exhalations which arife from thence, are feptic or promoters of putrefaction, has been fully demonftrated by the experiments of Dr. Alexander, of Edinburgh.

But that any exhalation or other fubftance, fhould act on the moving powers or folids of the human body, feveral days after it it has been received into the body, without making ſome material change in the condition or quality of the circulating fluids is inadmiſſible, becauſe it is ſcarcely conceivable. That ſuch alteration is made in the quality of the fluids in putrid fevers is manifeſt from the contagious effects of the ſeveral excretions. But in caſes of intermittents and remittents which originate in marſhy ſituations, no ſuch evidence is afforded, for there is no authentic inſtance of theſe being contagious or communicable from one to another.

As no other exhalations or noxious matters than thoſe which have now been enumerated, can be diſcovered in the moſt unſalutary atmoſphere of marſhes; as there is no ſource from whence any other noxious ſubſtance can be introduced into the atmoſphere of ſuch ſituations; and as it is evident from the known effects of the gaſes which have been diſcovered in it, that they cannot have the effect of producing the diſeaſes under conſideration either when applied ſingly or united, we certainly ought to heſitate before we adopt the doctrine heretofore taught, reſpecting marſh miaſma.

But as it is well known that a very material alteration is made in the proportions, which one of the component parts of the atmoſphere bears to the other, by certain proceſſes of nature and art, let us enquire how far the alteration which is made in the atmoſphere of marſhes, by the proceſs of putrefaction, may affect the preſent queſtion.

Mr. Vanbreda's experiments prove that there is leſs oxygen in the atmoſphere of marſhes during autumn, when the weather is dry and hot, than in more ſalutary ſituations; and it is well known, from innumerable experiments made by different philoſophers, that this can only be diminiſhed by combuſtion, fermentation, putrefaction, or reſpiration, or a proceſs of a ſimilar kind.

It is alſo a fact fully eſtabliſhed, that the functions of life, as well as the proceſs of combuſtion and fermentation, can only be continued by the application of oxygenous gas, and that theſe are affected in proportion to the quantity and purity of the gas applied.

It was formerly diſcovered by Veſalius, and has ſince been confirmed by the obſervations of Doctors Lower, Prieſtley, Crawford, and others, that the blood in the pulmonary veins is of as red and florid a colour as in the arteries, which is the reverſe in every other part of the ſyſtem. This circumſtance has been demonſtratively proved to be owing to the action of the oxygen, or the baſe of pure air upon the blood in the pulmonary veins.

From the experiments of the diſcerning and ingenious Dr. Goodwin upon living animals, it appears, that the action of the heart cannot be continued by the reception of blood, which has not undergone this change of colour in the pulmonary veins from the application or introduction of oxygen. This fact has been since confirmed by the experiments of Dr. Girtanner, as may be seen in his Essay on the Principle and Laws of Irritability.

That blood impregnated with oxygen, or the base of pure air, is the necessary and appropriate stimulus for giving motion to the heart, and enabling it to carry on the circulation of the blood, was rendered evident from the gradual diminution and debility of its contractions, as the colour of the blood became darker when the pure air was excluded, and from its contractions becoming stronger as the blood recovered its florid colour from the application of pure air.

In these experiments, all the other functions of the body were observed to be proportionally affected with the heart. As its constractions diminished, the power of these also declined; As the power of the heart recovered, these also recovered.

By these experiments, we learn that the abstraction or exclusion of the oxygenous part of the atmosphere, in a given space, is sufficient of itself to deprive animals of life, by withholding the cause of action. Hence we are authorised, by the chastest rules of induction, to conclude that health and life must be affected, more or less, in proportion to the quantity of this vivifying principle, at any time abstracted from the atmosphere, which more immediately surrounds us.

The presence of the other component part of the atmosphere, the base of the azotic gas, though totally opposite to the oxygen, with which it forms a perfect compound, and neutral substance, when mixed in the proportions already mentioned, appears to have no share in destroying life, though its name is derived from a mistaken supposition that it had that effect; for the heart immersed in this gas, will retain its irritability several hours, in a warm situation, after all signs of life have disappeared in the rest of the body. Mr. Valli's experiments on animal electricity have established this fact.

Carbonic gas or fixed air, on the contrary, produces its destructive effects by a direct operation, for it destroys the nervous power, and the irritability of the muscular fibres, the instant that it is received * into the lungs, and comes in contact with the heart.

If the carbonic gas operated, as suggested by Mr. Kite, by inducing a spasm of the glottis, and thereby excluding the atmospheric air, the heart, as in other cases of suspended respiration,

* This expression is not correct; see Mr. Davy's experiments. EDIT.

ation, would retain its irritability for some time; but this is not the case.

From the facts and observations which have now been stated, I think it may be fairly concluded, that the causes of the unwholesomeness of low and moist situations in the summer and autumnal months, is not owing to any invisible miasmata or noxious effluvia, which issue from the soil and lurk in the air, but to a very different cause, viz. to a deficiency of the oxygenous portion of the atmosphere in such situations, in consequence of vegetable and animal putrefaction, in conjunction with the exhausting and debilitating heat of the days, and the sedative power of the cold and damp air of the nights.

For want of the refreshing and salutary stimulus of pure air, all the functions of the body are performed imperfectly and languidly. The nervous system, in particular, becomes preternaturally susceptible of impressions from every change that occurs in the temperature of the surrounding atmosphere. The application of, or exposure to, a damper and colder state of the air than usual, renders the vessels on the surface of the body powerless, and atonic, the brain and heart sympathise with the extreme nerves and vessels, the power of every function of the body declines, till the heart, roused by accumulating blood, reacts with increasing velocity, and is relieved of the unusual burthen.

That the causes which I have now assigned are the true ones, is rendered next to certain, from the frequent occurrence of those diseases, (which have heretofore been supposed to depend upon the operation of specific miasmata) in situations remote from marshy ground, particularly in large and populous cities, where sedentary occupations and want of exercise, render the inhabitants delicate and infirm. I have seen numerous instances of this kind even in the winter season, when no effluvia from marshes could possibly exist, especially among those who had been previously debilitated by other disorders. Nor is it uncommon for persons who have recovered from intermittents in the autumn, to have frequent recurrences of the same disease in the winter, merely from sitting in a damp room, or other exposure to cold.

If the diseases of marshy situations were produced by a specific matter, they could never be produced by any other cause; but as they are frequently induced in seasons and situations where that supposed specific matter or miasma cannot possibly exist, there is nothing more clear than that they are not produced by any such specific matter.

The opinion that those diseases are the product of specific matter generated by vegetable putrefaction, appears to be rendered groundless from the disease varying in its type and symptoms, toms, in proportion to the extent and putridity of the soil, state of climate, season and weather with respect to heat, moisture, &c. and also in its not being contagious, the reverse of which is the case with all known diseases that are derived from specific matter.

We are assured by the accurate Monro, in his account of the diseases which prevailed in the military hospitals in Germany, in 1761 and 1762, that the intermittent fever seldom attacked any but those whose solids had been previously relaxed by the preceding heat of the summer, except when they had been fatigued and over-heated by the sun, and afterwards exposed to the evening dews.

Dr. Lind, of Windsor, says, sudden exposure to cold occasioned either an inflammatory fever, or a simple intermittent, at Bengal, according to the predisposition of the body.

The scurvy, as well as the diseases already enumerated, also appears to derive its existence from a deficiency of pure air in conjunction with a cold and moist atmosphere, and a diet of salted flesh meats; for it generally prevails in long voyages, after a continuance of wet weather. The hatches being kept shut at such times, prevents ventilation; in consequence of which the oxygen becomes exhausted.

Captain Cook, in his two last voyages, preserved his crew from the scurvy by frequent ventilation, constant cleanliness, suitable cloathing, and strict discipline.

Dr. Trotter assures us, that in a slave ship of which he was surgeon, the seamen that were constantly on deck, and fed with the ordinary sea diet, remained free from the scurvy, while the slaves that lived principally on vegetables, but breathed a confined impure air, fell miserable victims to it.

The remarkable case of the blue boy,* described by Dr. Sandifort of Leyden, furnishes another striking example of the importance of oxygen in the preservation of health and life, as well as a confirmation of its being the cause of the red colour of the blood.

In this boy, whose skin was as blue as indigo, the aorta communicated with both ventricles of the heart; in consequence of which the greatest part of the blood was immediately propelled from the right ventricle into the aorta, so that very little passed into the pulmonary artery to be oxygenated.

An opinion equally erroneous with that which has lately prevailed respecting the causes of intermitting fevers, &c. has also been delivered down from age to age, respecting the causes of continued fevers of the nervous or putrid kind.

The

* I have seen a similar instance. T. B.

The doctrine formerly taught refpecting thefe, was, that they derived their exiftence from the effluvia of dead and putrid animal fubftances: But from more recent and accurate obfervations, it appears that the contagion by which this kind of fever is produced, as well as thofe of a peftilential nature, is always derived from the living human body in confined and unventilated fituations; and it is probable that the effluvias thus excreted, partake of the quality of nitrogen gas, from their being rendered harmlefs by an union with oxygen or the bafe of pure air.

It appears more than probable alfo, from the hiftory of the circumftances always prefent at the time febrile contagion is generated, that it is rendered virulent and powerful, in proportion to the abfence or defect of oxygen, and the degree of heat to which the living body has been expofed in fuch fituations. It was a concurrence of thefe circumftances which gave origin to the yellow fever which appeared in Grenada in the beginning of the year 1793, and which was afterwards imported into Philadelphia, as appears from the account publifhed by Dr. Chifholm.*

Noxious effluvia indeed frequently arife from putrid animal fubftances in confined fituations. Dr. Monro mentions a remarkable inftance of this, and fome later examples are recorded by Mr. St. John; but it does not appear from thefe cafes, that thofe noxious effluvia produced any fymptoms refembling thofe of putrid or peftilential fevers; on the contrary, they acted as direct ftimulants, and occafioned inflammatory affections without being preceded by that fenfe of debility which always precedes thofe fevers that are occafioned by febrile contagion.

Having now fhewn that the difeafes which prevail moft generally during the autumnal feafon in low and marfhy fituations, owe their origin, not to invifible exhalations or miasmata, but to the caufes which I have affigned, the prophylaxis, or the means of preventing the occurrence of thofe difeafes, muft be fimple and obvious.

Thefe are to introduce and increafe the proportion of oxygenous gas in the fuperincumbent atmofphere, and to prevent its future abftraction, by cutting off or diminifhing the fources of putrefaction.

It would be a happy circumftance if the application of the means fuited to produce an amendment in a body fo large and fluctuating as the atmofphere, was as practicable as the means fuited to effect that purpofe are obvious: but unfortunately, this

* Vide Chifholm's Effay on the Fever of Grenada in 1793, &c.

this requires too much labour and expence to admit of extenſive application, eſpecially in a country where population and wealth do not bear a due proportion to the extent of territory.

We ought however to attempt every thing in our power to effect ſo deſirable and uſeful an event.

Chemiſtry furniſhes various articles by means of which we can generate and introduce a ſupply of oxygen into the atmoſphere, as well as alter the quality of thoſe noxious gaſes with which it is occaſionally contaminated.

Theſe however can only be employed in a very limited and partial manner, and of courſe can only produce a limited and partial amendment.

I ſhall therefore mention only a few of the ſubſtances that may be occaſionally employed for this purpoſe.

A large portion of oxygen may be furniſhed by the decompoſition of nitre, as is demonſtrated from its maintaining the combuſtion of inflammable bodies.

If lighted charcoal be placed in a proper expoſure to the open air, it will continue to burn till the whole be reduced to aſhes.

If nitre be mixed with charcoal, and when kindled placed in a cloſe veſſel, the combuſtion will continue as well as if expoſed to the open air; whereas, without the aſſiſtance of the nitre, the charcoal would be immediately extinguiſhed in that ſituation for want of a ſupply of oxygen.

Mr. Schele, by heating nitre to red heat in a retort, received into a moiſtened bladder more than fifty ounces in meaſure of oxygen gas from one ounce of nitre. A pound will therefore furniſh 800 ounces.

Nitre ground with two-thirds of its weight of minium, and moiſtened with water ſo as to form a paſte, burns very rapidly, and emits a conſiderable quantity of pure air.

But the grand engine by which the ſources that deprive the atmoſphere of its ſalutary and vivifying principle, are to be cut off; and the great magazine, from whence a ſufficient ſupply is to be obtained, muſt be ſought for in the art of agriculture.

The ſtagnant waters may be carried off, and the ſoil of marſhes rendered dry, by means of drains, deep trenches, and wells; and farther ſtagnation and putrefaction prevented, by conſuming the dead weeds, graſs, and woods, and by filling up the flats, ſinks and hollows with clay, ſand, or lime.

And the atmoſphere may be ſupplied with a profuſion of oxygen, by cultivating on ſuch ſoils, graſſes and plants of vigorous growth, and eſpecially thoſe which live and flouriſh lateſt in the ſeaſon. For vegetables, while living and growing, when expoſed to the rays of light, conſtantly decompoſe the water they imbibe from the earth and air, and while they retain retain the hydrogen or base of inflammable air for the formation of oil, wax, honey, or resin, they replenish the atmosphere with oxygen."*

When it is impracticable to render marshy situations dry, on account of their extent, they should be kept constantly flooded by means of dams and sluices, to prevent the effects of putrefaction, for when dead vegetable or animal substances are immersed in water so as to be entirely excluded from contact with the air, putrefaction can only take place in a slow and imperfect manner.

But clearing the woods, plants, and herbs, from marshy or fenny tracts without draining off the stagnant water at the same time, and destroying the dead herbage by fire, instead of rendering such situations more healthful has been found to have a different effect, because a greater extent of putrescent surface is thereby exposed to the rays of the sun, and of course a greater portion of oxygen abstracted from the atmosphere. It is owing in a great measure to this circumstance, that all new countries are so generally fatal to the first settlers.

The same land after it has been cultivated a few years, especially if there be sufficient declivity to prevent the water from stagnating, loses its unwholesomeness, the putrescent substances mixed with the soil or superficial stratum of the ground having finished the putrefactive process by that time. In order therefore to render and preserve marshy countries healthful, they should be preserved dry and clean by means of the spade, the plough, and the rake.

When the level situation of a place prevents the stagnant water from being carried off by drains, deep wells should be dug, in different places, for the water to collect in, by which means a greater portion of the soil will be rendered dry, and less noxious.

To prevent still farther the injurious effects of residing near marshes or mill-ponds; rows of such trees as grow rapidly, and retain their verdure late in the season, should be planted between those situations and the mansion, for the purpose of intercepting the moisture in its progress, while they furnish a constant supply of oxygen to the atmosphere.

Lodging in the upper story of a house has been found to preserve health during a sickly season, instances of which are recorded by Sir John Pringle. This appears to be owing to those situations being out of the reach of the moisture from the ground.

---

* Chaptal's Chemistry. Ingenhousz's Observations, &c.

